# Assessing myelofibrosis burden on QoL and productivity from healthcare personnel and patient perspectives in India

**DOI:** 10.1186/s12885-025-14324-4

**Published:** 2025-07-01

**Authors:** Prantar Chakrabarti, Abhay Bhave, Claire Harrison, Tulika Seth, Vikram Mathews, Disha Shetty

**Affiliations:** 1 Zoho Corporation, Consultant Haematologist, Chennai, India; 2 Empire Centre Haematology & Onocology Speciality Clinic, Mumbai, India; 3 Guy’s and St Thomas’ NHS Foundation Trust, London, UK; 4 AIIMS, New Delhi, India; 5 Christian Medical College, Vellore, India; 6 Franchise Medical Head- Oncology, Novartis Healthcare Private Limited India, Mumbai, India

**Keywords:** Myelofibrosis, Myeloproliferative neoplasms, Symptom burden, Quality of life

## Abstract

**Background:**

Myelofibrosis (MF), leads to variety of symptoms, significantly impacting patients’ quality of life (QoL) and work productivity. Limited data exists on MF in India. This survey conducted among Indian physicians and their patients with MF evaluated patient journey, understand perceptions around symptom burden, its impact on QoL, and management.

**Methods:**

This cross-sectional and multicentric survey between October to November 2021 across 17 cities was conducted using a structured questionnaire based on certain identified themes (symptom burden, QoL parameters, treatment goals and patient communication) during one-on-one telephonic interview. Descriptive statistics were used for data analysis.

**Results:**

Fifty physicians and 154 patients (primary MF: 51, post-PV: 78, post-ET: 25) participated. Diagnostic delay of 10 months from symptom onset was noted. 151 (98%) patients reported reduced QoL and loss of work productivity. Majority of the patients (56%) reported a negative impact on work productivity, with 76% confirming that MF hindered their work-related daily activities and 42% having reduced work hours. Key symptoms included abdominal pain (patients: 81%, physicians: 70%), fatigue (77% vs. 73%), and fever (54% vs. 48%). Patient-physician perception discrepancies on QoL factors were notable, particularly bone pain (12% vs. 64%), sleep difficulties (8% vs. 70%), sexual problems (2% vs. 78%), depression (4% vs. 63%), and inactivity (4% vs. 66%).

**Conclusion:**

The study results highlight the negative impact of MF on work productivity by hampering their work-related daily activities and reducing the work hours. The findings underscore the significance of enhancing patient-physician communication and standardizing monitoring practices for improving care outcomes.

**Supplementary Information:**

The online version contains supplementary material available at 10.1186/s12885-025-14324-4.

## Background

Myeloproliferative neoplasms (MPNs) are clonal or oligoclonal disorders caused by over proliferation of the bone marrow stem cells [1]. Patients with classical Philadelphia-negative MPNs (Myelofibrosis, Polycythemia Vera, and Essential Thrombocythemia) have a time dependent risk of progression to either an advanced myelofibrotic state (post ET/PV MF) and /or to acute myeloid leukemia. MF represents the most severe type of classic Philadelphia chromosome-negative MPNs which is characterized by stem cell-derived clonal myeloproliferation and anomalous production of cytokines [2]. Complications of MF include early satiety, progressive splenomegaly, hepatomegaly, portal hypertension, and thromboembolic events, involving both microvascular and macrovascular issues such as atherosclerotic, reno-vascular, cardiac, and cerebral vasculopathies, as well as transient ischemic stroke (TIA), deep vein thrombosis (DVT), and peripheral vascular disease (PVD). Additionally, cytopenia, transfusion burden, infection risk, and potential leukemic transformation contribute to reduced patient survival [3–6]. Patients may experience a broad spectrum of symptoms, ranging from mild to debilitating, including fatigue, bone pain, weight loss, pruritus, numbness in the hands or feet, and night sweats. More severe symptoms can include abnormal bleeding, sudden abdominal pain, shortness of breath, sudden chest pain, and facial flushing. These symptoms can significantly impact their quality of life (QoL), affecting both their physical and psychosocial well-being with challenges like depression and anxiety [7]. The disease’s unpredictability and symptoms, along with regular treatments, can limit social interactions, leading to isolation due to fatigue and discomfort.

The long-term nature of MPNs, coupled with the need for continuous treatment, can result in substantial medical costs. Frequent healthcare visits can lead to indirect costs like transportation and lost wages, while also reducing work productivity and causing extended absences. While previous research studies have addressed the challenges of managing MF in foreign settings, there was limited data on how these factors impact patients in India. This makes this research paper the first to evaluate these aspects in Indian MF patients. The present strategy for managing MF relies on a symptomatic approach [2].

Limited data is available concerning patient and physician perceptions about MPN symptomatology and treatment goals from the Indian setting. Given the cultural, socioeconomic, and healthcare differences in India compared to Western countries, it is crucial to understand both patient and physician perspectives on the disease’s impact on QoL. Attempts to better understand these perceptions may lead to optimized patient care and, ultimately, better patient outcomes.

Many clinical trials have reported improved survival rates with the use of novel treatment strategies in patients with MF [2, 8–10]. This study aims to assess both patient and physician understanding of the MF symptomatology and unmet needs during MF management in Indian clinics, while comparing their perceptions of symptoms and QoL. It also seeks to identify potential disparities between the perceptions of patients and physicians by highlighting the unique challenges and experiences of Indian MF patients and their treating physicians.

## Methodology

### Study design and survey instrument

The current cross-sectional and multicentric survey involving patients with MF and their treating physicians was conducted during October 2021 to November 2021 across the four zones (East, North, South, and West) in 17 cities. A total of 50 physicians, including haematologists/ haemato-oncologists and medical oncologists, participated in the study. Additionally, 154 patients, recruited by these 50 healthcare professionals, completed a separate questionnaire [Supplementary Appendices 1, 2 and 3]. The survey questionnaire, a multi-item construct, was designed in English in consultation with the subject matter expert and patient caregiver in line with the published Landmark MPN survey, other global MPN surveys, and known symptom assessment form [5, 11–14]. The questionnaire was prepared considering the objectives of the study and subjected to review by the study investigators. The information collected from the patient and physician surveys were primarily related to patient journey, symptomatology, diagnostic attributes, impact on QoL, work-related activities, treatment goals, practices, satisfaction with the treatment, in-clinic communication, and patient satisfaction. The two components of the survey (patient and physician surveys) were conducted separately. One-on-one telephonic interview with the physicians and patients were conducted using the Computer-Assisted Personal Interviewing (CAPI) technique. The interviews lasted for not more than 40 min. The responses were assessed using a 10-point scale. This questionnaire helped capture the differences in perceptions between physicians and patients throughout the patient journey. To ensure the quality and consistency of the data collected, training interviewers on standardized probing techniques and using clear and unambiguous language in the interview process across participants was undertaken.

### Study population

The physician recruitment was done using telephonic screening by the field staff where haematologists, medical oncologists, and haemato-oncologists across the four zones were targeted. The physicians who agreed to audio recording and getting re-contacted in case of query were interviewed based on screening questions. The screening criteria for physicians included type of practice (private or public practice), total number of years of practice (range: 3–30 years, terminated if less than 3 years and more than 30 years) and number of patients with MF under the care in the past 12 months (terminated if less than 3). Based on the responses provided by the physician, the main interview for the respective physician was scheduled.

The physicians included in the study recruited the patients from their patient pool based on the screening criteria. The screening criteria for patients included patient’s age (> 18 years), duration of diagnosis with MF (> 6 months ago) and consulting physician (haematologist, haemato-oncologist, or medical oncologist). The patients who provided their consent to processing of personal data agreed to audio recording of the interview and re-contacted in case of query continued with the questionnaire. The target respondents under the patient category were further segregated based on disease type (primary, post-PV and post-ET MF) and geography (metro vs. non-metro).

### Ethics consideration

This survey was approved by the Royal Pune Independent Ethics Committee, Pune, Maharashtra, India (DCGI Registration No: ECR/45/Indt/MH/2013/RR-19; Approval No: RPIEC050921). The IEC members reviewing the survey were independent from the investigators and sponsor. The research procedure was in accordance with the ethical standards of the IEC, the national guidelines, and Market Research Society’s Code of Conduct and adhered to the guidelines in the Declaration of Helsinki. The informed consent was obtained electronically from the survey participants before inclusion in the study.

### Statistical analysis

The reported statistics were based on the type of variables described. For numerical variables, the respondent base, mean, percentages and range (minimum and maximum values) were reported. Additionally, the responses obtained on a 10-point scale were converted to percentages for representation. The T3B (Top 3 Boxes) scores were employed in the study. Respondents were asked to rate parameters on a scale of 1 to 10. T3B refers to the proportion of physicians who gave a rating of 8, 9 or 10. For instance, if the sample size is 100 and the T3B is 80%, then it means 80 out of 100 physicians gave a rating of 8, 9 or 10.

For categorical variables, the total number of respondents and the number and percentage of responses are reported. Sub-group analyses included patient category (primary MF, post-PV, and post-ET), risk category, SEC (socioeconomic class), metro versus non-metro cities, and insured versus non-insured patients.

To assess the statistical significance of the difference between physician and patients’ perception towards different patient-related outcomes, one-sample z-tests for proportions were conducted. In these tests, the mean of the physicians’ estimates for each symptom and QoL indicator served as the hypothesized population proportion, against which the corresponding observed proportion in the patient group was compared. The correlation between the MPN-10 and DIPSS score was determined using Pearson’s correlation test.

## Results

### Patient demographics

Out of the total 154 patients, 33%, 51% and 16% were diagnosed with primary MF, post-PV, and post-ET, respectively. The average age of the patients interviewed was 51 years (range: 25–80 years), with a higher representation of male patients (68%). Majority of the patients (99%) in the study belonged to the SEC A category, which was defined as the uppermost segment of the consuming class with an average annual household income of INR 7.5 lakhs (9370 USD) [15].

On an average, patients were diagnosed with MF at the age of 49 years (range: 22–79 years). A total of 8%, 33%, 23% and 36% of patients had low, intermediate-1 risk, intermediate-2 risk and high risk respectively, as per the Dynamic International Prognostic Scoring System (DIPSS). The study found that for 98% of the sample, the average time from initial diagnosis to inclusion in the study was 1.5 years, with a range of 1 to 4 years. Patients of different risk categories were further stratified into quartiles based on their total symptom score. The severity of symptom that patients suffer from is not dictated by the risk categorization. Significant correction was not observed between severity of symptoms as per MPN-10 and disease severity as per DIPSS score (*p*˃0.05).” Table 1.

**Table 1:** MPN-10 quartiles as per risk categories

MPN-10 Quartiles as per Risk Categories				
Proportion split of patients across Quartiles	0%	5%	37%	58%
Risk catefory as per dipss parameters	Q1 *n* (%)	Q2 *n* (%)	Q3 *n* (%)	Q4 *n* (%)
Low Risk [Avg. MPN-10 Score:79] (*n* = 13)	0 (0%)	1 (8%)	1 (8%)	11 (85%)
Intermediate 1 Risk [Avg. MPN-10 Score:72] (*n* = 51)	0 (0%)	3 (6%)	22.9 (45%)	24.9 (49%)
Intermediate 2 Risk [Avg. MPN-10 Score:71] (*n* = 35)	0 (0%)	2.1 (6%)	17.8 (51%)	15 (43%)
High Risk [Avg. MPN-10 Score:78] (*n* = 55)	0 (0%)	2.2 (4%)	14.8 (27%)	37.95 (69%)

MPN-Myeloproliferative neoplasms

### Physician demographics

Majority of the physicians (64%) were haematologists, while 20% were medical oncologists and 16% were haemato-oncologists, who were predominantly practicing in a private setting. The physicians had an average experience of 16 years (range: 3–30 years) and consulted an average of 14 patients with MF in the past 12 months (range: 3–60 patients).

### Patient symptomatology

The most common symptoms experienced by patients with MF at the time of diagnosis, as reported by patients and physicians, were abdominal pain/discomfort (81% vs. 70%), fatigue/tiredness/weakness (77% vs. 73%) and fever (54% vs. 48%) Table 2.

**Table 2:** Patient symptomology

	Overall		Primary MF		Post PV MF		Post ET MF	
Symptoms	Physician (*N* = 50) n (%)	Patient (*N* = 154) n (%)	Physician (*N* = 50) n (%)	Patient (*N* = 51) n (%)	Physician (*N* = 50) n (%)	Patient (*N* = 78) *n* (%)	Physician (*N* = 50) n (%)	Patient (*N* = 25) n (%)
Fatigue/Weakness/Tiredness	37 (73)	119 (77)	37 (73)	40 (79)	37 (73)	59 (75)	37 (73)	20 (78)
Abdominal Pain/Discomfort	35 (70)	125 (81)	35 (70)	39 (77)	35 (70)	66 (85)	35 (70)	19 (77)
Fever	24 (48)	83 (54)	24 (48)	24 (47)	24 (48)	46 (59)	24 (48)	13 (52)

The most common symptoms at diagnosis continued to be the symptoms patients faced during the study period, including abdominal pain (92% at diagnosis vs. 77% currently), fatigue/tiredness (77% vs. 71%), weakness (77% vs. 75%), abdominal discomfort (64% vs. 51%), and headache (35% vs. 28%).

Abdominal pain/discomfort was observed more frequently in post PV MF patients (85%) than other subtypes (77%). Headaches manifested more commonly in Post ET patients (36%). Differences existed among Post ET patients who faced difficulty sleeping and Post PV patients who commonly encountered bone pain.

### Differences in physician and patient perceptions on symptomatology and impact on QoL

**Fig. 1:**
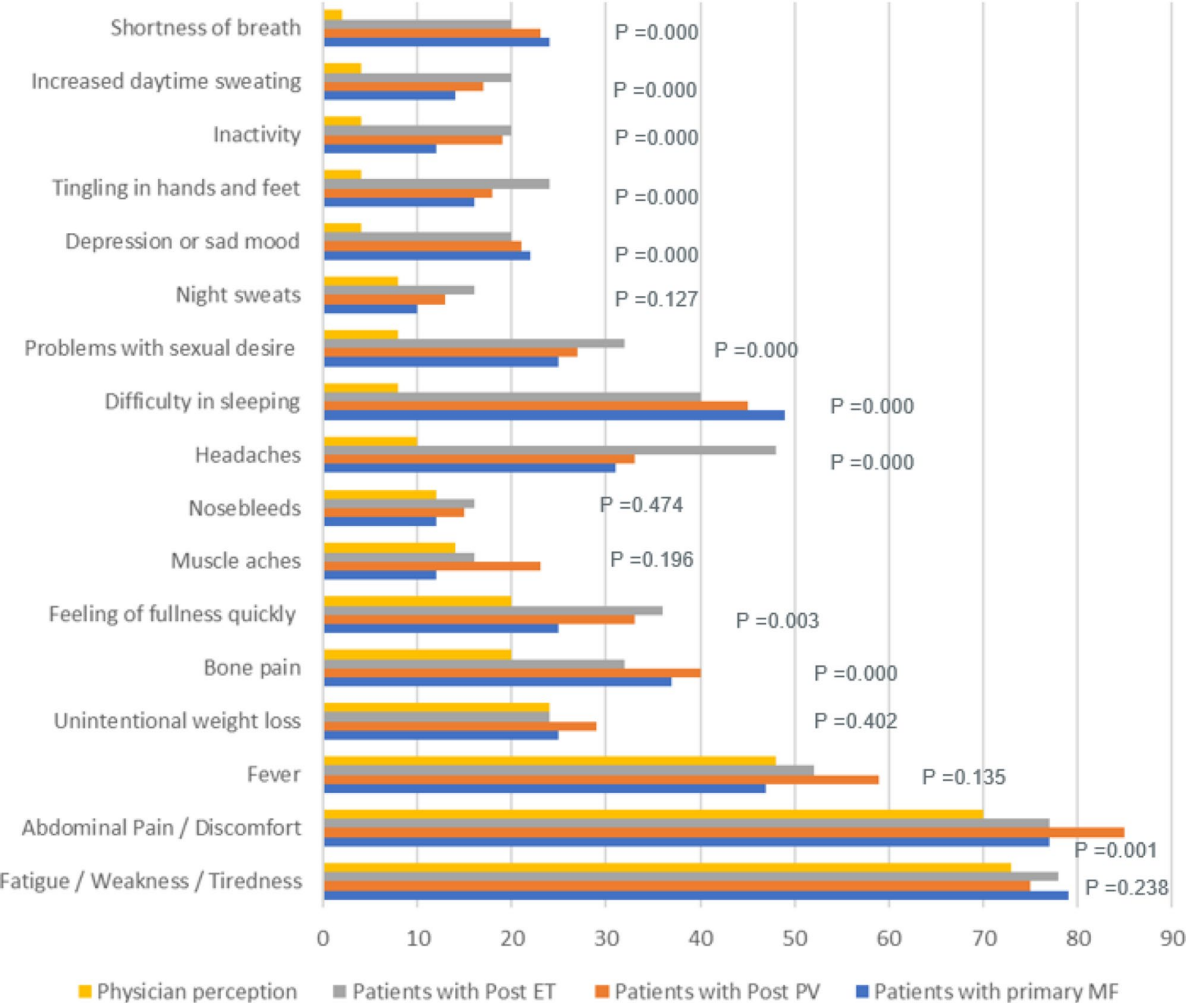
Patient’s (Primary MF, Post-PV, Post-ET) And Physician’s Perceptions on Symptom Burden. Abbreviations: ET- essential thrombocythemia; MF- myelofibrosis; PV- polycythemia vera. Note: The difference in patient and physician perception on symptom burden was statistically not significant for fatigue / weakness / tiredness, fever, unintentional weight loss, muscle aches, nosebleeds, and night sweats

While both physicians and patients agreed that most frequent symptoms observed were fatigue/ weakness, abdominal pain/ discomfort and fever, the highest disconnect was observed in some parameters where more patients considered difficulty in sleeping (45% vs. 8%), headaches (35% vs. 10%), bone pain (38% vs. 20%), problems with sexual desire (27% vs. 8%) and shortness of breath (23% vs. 2%) as significant issues as compared to their treating physicians. The differences between patient and physician responses on symptom burden were assessed and has been represented in Fig. 1. The *p* values have been calculated to determine whether the difference is statistically significant or not (*p*˂0.05, 95% CI) Fig. 1.

**Fig. 2:**
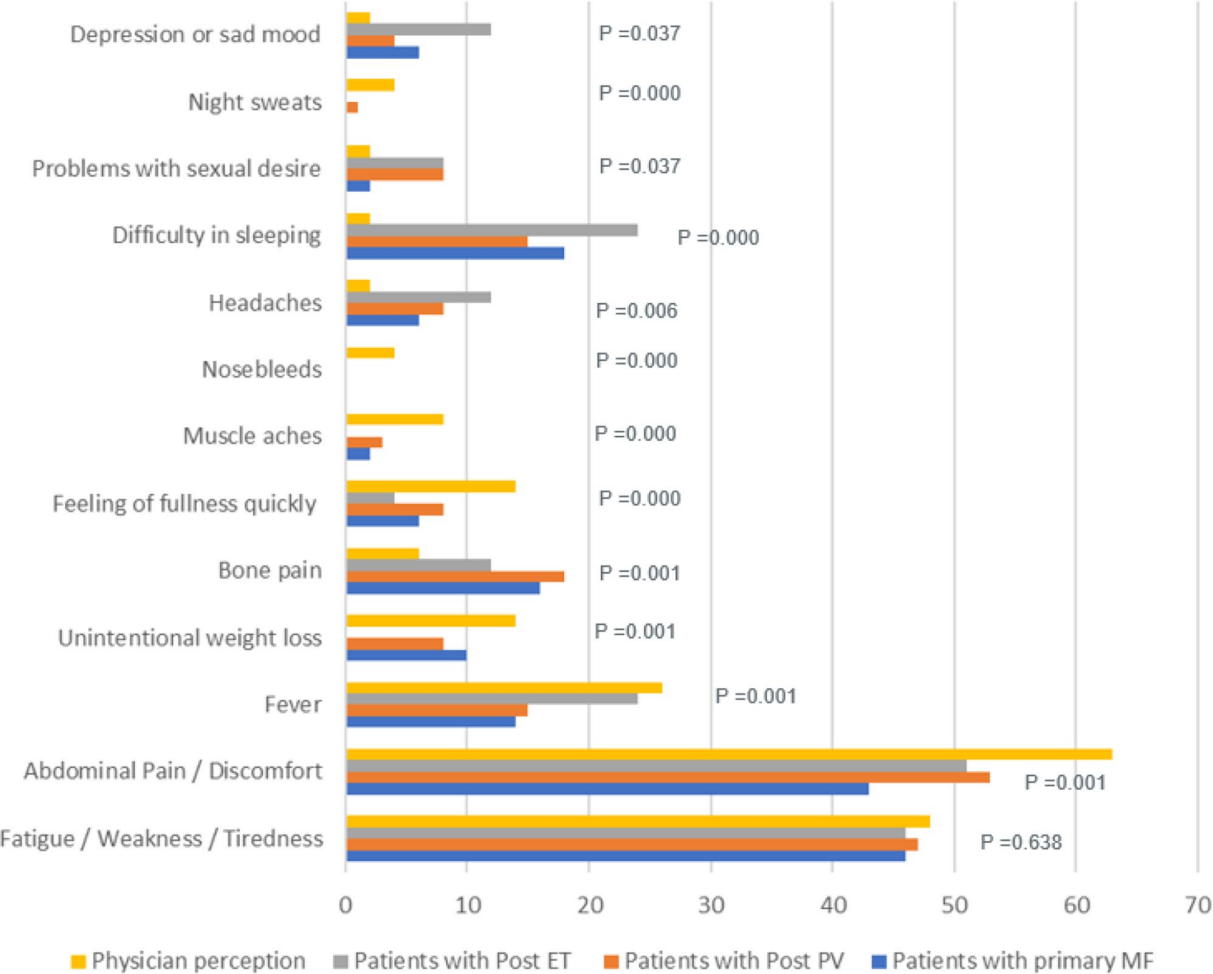
Patient (Primary MF, Post-PV and Post-ET) And Physician Perceptions on Symptoms Requiring Early Resolution. Abbreviations: ET- essential thrombocythemia; MF- myelofibrosis; PV- polycythemia vera. Note: The difference in patient and physician perception was statistically not significant for fatigue / weakness / tiredness

Physician and patient perceptions differed on which symptoms required early resolution. Physicians primarily focused on a few key symptoms, such as abdominal pain/discomfort (63%), fatigue (48%), and fever (26%). However, patients, beyond these symptoms, also expected other issues like bone pain and difficulty in sleeping to be resolved, which were not considered a priority by physicians. For symptoms requiring resolution, the difference in patient and physician perception was statistically significant for all except fatigue (*p*˃0.05, 95% CI) Fig. 2.

**Fig. 3:**
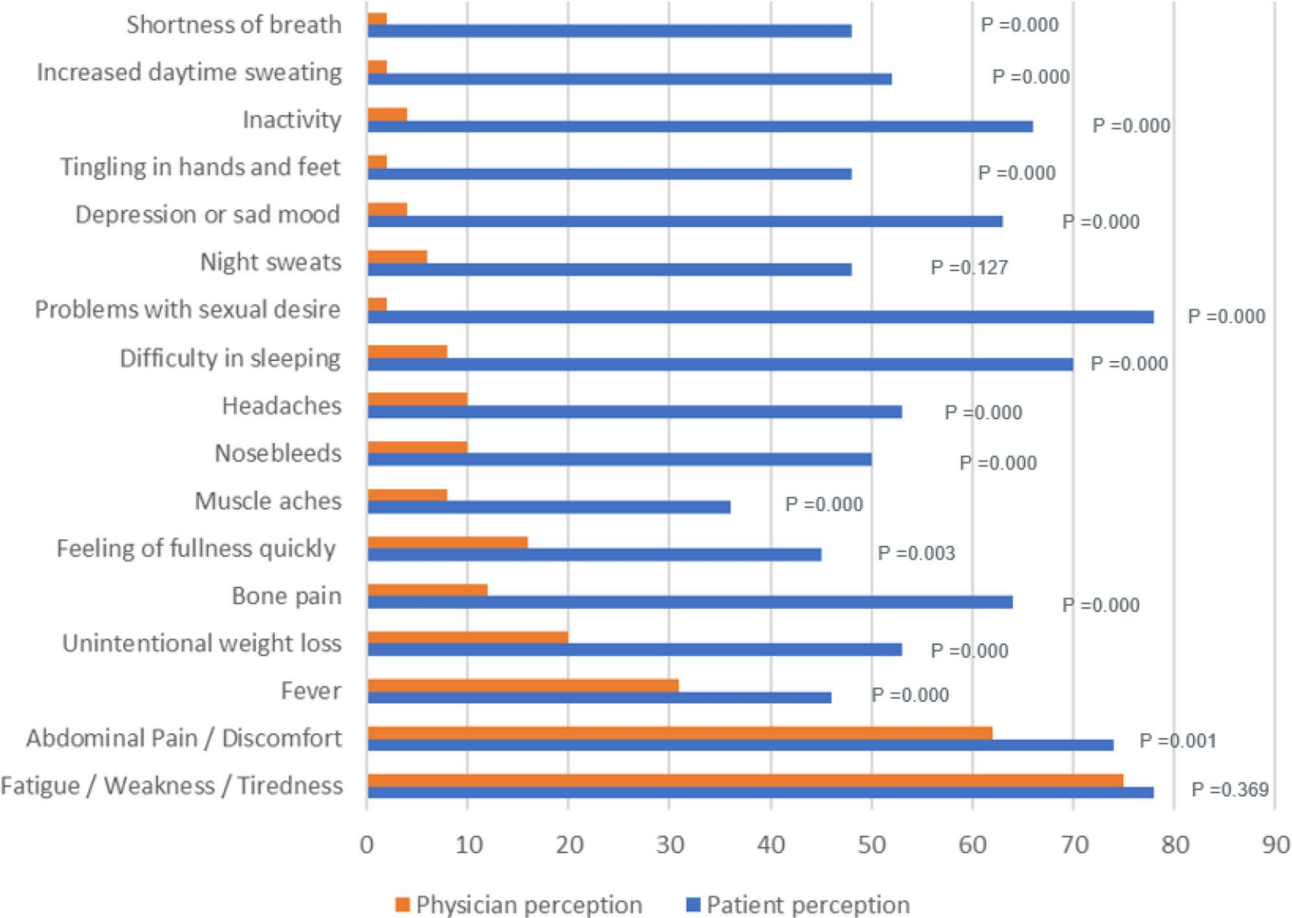
Patient and Physician Perceptions on Symptoms Negatively Impacting Quality of Life of the Patients. Note: Difference between responses of patient and physician on negative impact of symptoms on quality of life were statistically significant for all the symptoms except fatigue / weakness / tiredness

The common symptoms reported by the physicians and patients that adversely impacted the QoL of the patients were fatigue (75% vs. 78%), abdominal pain (62% vs. 74%) and fever (31% vs. 46%). A stark difference in the perception of QoL was noted between the physicians and the patients regarding parameters such as bone pain (12% vs. 64%), difficulty in sleeping (8% vs. 70%), sexual problems (2% vs. 78%), depression (4% vs. 63%), and inactivity (4% vs. 66%) respectively. The difference in perception was statistically not significant for fatigue (*p*˃0.05, 95% CI) Fig. 3.

### Treatment goals

More than 60% of physicians considered better quality of life (88%) and laboratory parameters such as anemia correction (62%), healthy blood counts correction (66%) as the main goal of treatment. This differed from patient expectations who considered symptom improvement (69% patients vs. 12% physicians) as their main treatment goal, in addition to better quality of life (74%). Patients considered feedback from physicians (82%) as the most important indicator to determine success of MF treatment, followed by the enhancement in QoL (44%) and fewer MF symptoms (44%).

### Assessment of MF symptoms

Majority of the physicians (80%) felt that they were proactive while assessing patient’s symptoms. However, patients believed the opposite that the physician waited for them to talk about their symptoms. Additionally, 54% of the physician confirmed on the regular use of MPN-10 tool for symptom assessment of patients in their clinic while only 1% of the surveyed patients agreed that their physician recommended the use of the MPN-10 assessment tool.

### In-clinic communication and impact on patient Understanding of disease

Over 98% of physicians are aware that myelofibrosis is a blood cancer that can progress to serious outcomes and significantly impact patients’ quality of life. However, only 26% of physicians discuss myelofibrosis as a cancer with their patients. Most of the patient (64%) counsel that it is a manageable blood disorder, and only 10% inform them that the condition will require lifelong treatment. The impact on patient understanding is that over 90% of patients recognize myelofibrosis as a serious health condition that may require hospitalization and blood transfusions. However, none of the patients are aware that MF is a blood cancer requiring lifelong treatment, which could negatively affect treatment adherence in clinical settings.

Both physicians and patients agree that more time is spent during consultations at the time of diagnosis (20–30 min), but less time is allocated during follow-up visits. Additionally, 94% of patients feel that the consultation time is insufficient to discuss all their symptoms. Furthermore, 91% of patients believe that physicians primarily focusses on blood count assessments rather than addressing symptoms during consultations. Over 70% of patients feel that symptoms are only discussed when their condition is severe, and more often, they discuss their symptoms with nurses and other paramedical staff at the clinic Table 3.

**Table 3:** Physician and patient perceptions on the disease Understanding and In-clinic communication

**Physician’s Perception on MF**	**Physician’s Response**		
	**Overall n (%)** **(n = 50)**	**Metro n (%**) **(n = 29)**	**Non-metro n (%)** **(n = 21)**
Myelofibrosis is a blood cancer	49 (98%)	29 (100%)	19.9 (95%)
Myelofibrosis may progress to a more serious condition	50 (100%)	29 (100%)	21 (100%)
Myelofibrosis symptoms reduce a patient’s quality of life	50 (100%)	29 (100%)	21 (100%)
Reimbursement of drug treatments plays an important role in the treatment I offer to my myelofibrosis patients and/or is accepted by my patients	49 (98%)	28.1 (97%)	21 (100%)
**Physician In-clinic Communication About MF**			
The disease is discussed as a condition that may progress to cancer	25 (50%)	15.9 (55%)	9 (43%)
The disease and management are discussed as cancer	13 (26%)	6 (21%)	6.9 (33%)
It is discussed as a blood disorder that can be managed	7 (14%)	4.9 (17%)	2.1 (10%)
It is discussed as a condition that needs lifelong treatment	5 (10%)	2 (7%)	2.9 (14%)
**Patient Understanding of MF**	**Patient Response**		
	**Overall n (%)** **(n = 154)**	**Metro n (%)** **(n = 91)**	**Non-metro n (%) (n = 63)**
Myelofibrosis is a serious health condition	141.6 (92%)	91 (100%)	49.7 (79%)
Myelofibrosis may increase my hospitalization and transfusion needs	147.8 (96%)	91 (100%)	56.7 (90%)
Myelofibrosis may progress to a more serious condition	146.3 (95%)	91 (100%)	56 (89%)
Myelofibrosis symptoms reduce my life quality	150.9 (98%)	91 (100%)	59.8 (95%)

MF- Myelofibrosis; QoL- Quality of life

### Impact of disease on daily living of patients and emotional status of patients

While physicians recognized that MF affected patients’ daily activities, more physicians placed greater emphasis on physical hardships (96%) compared to emotional hardships (82%). In contrast, more patients attributed significance to emotional hardships (92%). Uncertainties surrounding their consultations (82%), frustrations regarding symptoms (81%), feelings of nervousness (80%), and sadness (79%) constituted the range of emotional distress experienced by patients with MF. For impact of MF on QoL, the difference in patient and physician responses were assessed and *p* values have been presented in Table 4 (*p*˃0.05, 95% CI).

**Table 4:** Impact on daily living due to myelofibrosis

	Physician Response (%) *N* = 50	Patient Response (%) *N* = 154	*p* values
**Impact on QoL Parameters (%))**			
Physical hardship	**96**	**78**	0.000
Sleeping habits have negatively changed	88	87	0.712
Irritable/angry and have not controlled it well	**86**	**76**	0.004
Condition is controlling their life	86	81	0.114
Depressed	86	77	0.008
Change in their appetite	86	86	1.000
Trouble coping with stress	86	77	0.008
Emotional hardship	86	92	0.006
Felt discouraged	82	79	0.361
Financial hardship	80	74	0.090
**Impact on Daily Activities (%)**			
Pain and discomfort have limited their activities	92	82	0.001
Condition has interfered with daily activities	86	76	0.004
Condition has interfered with family and social life	86	79	0.033
Condition has interfered with the relationship with their caregiver	84	77	0.039
Condition has interfered with sex life	74	66	0.036
**Emotional Status of the Patients (%)**			
I am unsure if I am being assessed and treated properly	NA	82	NA
I am frustrated tolerating the symptoms of my disease	NA	81	NA
I feel nervous	NA	80	NA
I feel sad	NA	79	NA
I am unsatisfied with how I am coping with my illness	NA	79	NA
I worry about dying/worry that my condition will get worse	NA	79	NA
I often feel worse than my physician is aware of	NA	78	NA
I feel frustrated having a rare, long-term illness	NA	77	NA
I feel helpless because of my disease	NA	77	NA
I am losing hope in the fight against my illness	NA	74	NA

NA- Not applicable

Note: For all the parameters where *p*˃0.05, no statistical difference was observed

Emotional burden was similar across risk categories and age groups of patients. Impact on daily living was also observed as the patients were forced to stay in bed all day, on an average of 3 days per month (range: 0–20 days), due to severity of the symptoms.

### Impact on productivity

Out of 154 patients, 55% (*n* = 85) were employed, and 45% (*n* = 69) were unemployed. Of the unemployed 53% (*n* = 37/69) were homemakers followed by 19% (*n* = 13/69) retired patients. Of the 85 patients working for pay, 51% (*n* = 43) were full employees, 2% (*n* = 2) were part time employees and 47% (*n* = 40) were self-employed. Nearly 56% of the employed patients reported a negative impact on work productivity, while 76% confirmed that MF hampered their work-related daily activities. A total of 42% of the patients reduced their hours at work, which on an average were cut back to 12 h per week (range: 1–28 h per week) Table 5.

A positive correlation has been observed between proportion of patients experiencing symptoms such as fatigue and the proportion of patients reporting a reduction in working hours. The *p* value was much higher than 0.05 for all symptom types and productivity parameters which indicates that correlation is statistically not significant.

**Table 5:** Percentage of patients with reduced hours of work

Employment situation	Overall (*N* = 154)		Metro (*N* = 91)		Non-metro (*N* = 63)	
% Patients	Yes n (%)	No n (%)	Yes n (%)	No n (%)	Yes n (%)	No n (%)
**Reduced your hours at work**	65 (42%)	89 (58%)	38 (42%)	53 (58%)	20 (32%)	43 (68%)
Gone on disability living allowance	5 (3%)	149(97%)	3 (3%)	88 (97%)	1 (2%)	62 (98%)
Taken a lower paid job	5 (3%)	149(97%)	2 (2%)	89 (98%)	2 (3%)	61 (97%)
Voluntarily terminated your job	3 (2%)	151(98%)	2 (2%)	89 (98%)	1 (2%)	62 (98%)
Taken early retirement	2 (1%)	153(99%)	2 (2%)	89 (98%)	0	63 (100%)
Been involuntarily terminated from your job	2 (1%)	153(99%)	1 (1%)	90 (99%)	0	63 (100%)

On an average, employed patients called in sick at work for 3 days (range: 1–12 days) out of the last 30 days due to MF-associated problems. MF significantly impacted caregivers’ daily life and productivity. Most caregivers were spouses/partners (64%) or children (31%). They spent an average of 9 h (range: 1–35 h) helping MF patients, mainly with transportation (75%), personal care (47%), emotional support (46%), and homemaking (46%). Most caregivers were not employed (89%), and those employed had to reduce work hours (73%) or switch to part-time (27%). Overall, patients reported a moderate impact on caregivers’ QoL. Both patients and physicians acknowledge that MF symptoms significantly affect QoL; however, physicians tend to focus more on the physical impact, while patients place equal importance on both the physical and emotional aspects. Low satisfaction with treatment leads to frustration, anxiety, and uncertainty regarding their diagnosis and treatment choices. This highlights the need for a more holistic approach to MF management and a multidisciplinary approach to patient care and counseling. The impact of the disease extends beyond the patients themselves, affecting their caregivers and family relationships as well.

## Discussion

The study demonstrates the perspectives of patients and physicians on the impact of MF on the QoL in Indian context. Notably, there is a dearth of research in India specifically addressing this aspect. This study offers valuable insights into the disease’s impact on patients’ emotional well-being and highlights the disparities in perspectives between patients and physicians, primarily due to lack of effective communication. The data suggests that the symptom burden is high, with a significant impact on patient QoL, notably on their physical and emotional well-being.

Our study reported that, on average, patients were diagnosed with MF at the age of 49, which aligns with the age range (47–66 years) reported in global studies. Among all risk categories, the highest proportion of patients were present in Q3 and Q4. Q4 represented most of the low-risk (85%), intermediate-1 risk (49%) and high risk (69%) patients, whereas Q3 represented majority of intermediate-2 risk (51%) patients, which is in alignment with other MPN surveys [12, 13, 16, 17]. Lower-risk patients may also experience a significant symptom burden, highlighting the high symptom burden among Indian MF patients which is similar to the findings in the global studies [12].

Abdominal pain (81%), fatigue (77%) and fever (51%) were the most experienced symptoms at the time of diagnosis. Differences in the perception of symptoms between the patient and physician in the current study was compared to the reported literature. Studies have demonstrated that 5–15% of patients complained of physicians either being uninterested or not asking about the symptoms [14]. In the German study group of MPN, weight loss was reported by the physician as a major symptom, which the patients did not consider associated with MF. This was in contrary to our study where weight loss was not considered a major symptom [18]. In the current study, physicians and patients aligned on fatigue being a well-recognized symptom in India, which differed from the Western literature. Other than fatigue, patient and physician perceptions differed over those symptoms requiring early resolution as the physicians focused on a small set of symptoms, but the patients expected a larger list of symptoms to be resolved. Abdominal pain/ discomfort and other symptoms were not regularly discussed by the in-clinic physicians. This indicated that many symptoms were left undiscussed which might have an impact on QoL and patient’s satisfaction with the therapies. Additionally, the recall from physicians on the number of symptoms is much lesser than patients with MF. It was hypothesized that splenomegaly and constitutional symptoms were high in the physicians’ mind and frequently touched upon in the clinics. However, the larger spectrum of symptoms that patients suffered from like bone pain, difficulty in sleeping, headaches, problems with sexual desire, inactivity and depression were assigned less weightage by the physicians.

Numerous MPN surveys revealed a comparable symptom burden: a survey carried out in the United States found that individuals with MPN mostly reported difficulty in sleeping and gastrointestinal discomfort [13]. The International MPN Landmark survey concluded that fatigue and abdominal pain were the most frequently occurring symptom in patients with MPN [12] which were underrecognized by the physicians globally. However, in our study, both the physicians and the patients reported fatigue as a well-recognized symptom. The Landmark survey from Taiwan also confirmed on fatigue being the most common symptom in patients with MF [17]. Fatigue, inactivity, and abdominal comfort were identified as the most common symptoms in MF patients in a retrospective review carried out in a tertiary healthcare environment in India, which was consistent with the symptoms reported by the patients in the current study [19]. Even though Indian physicians recognize fatigue as a common issue in MPN, their focus on other symptoms is minimal due to the lower use of objective assessment scales in India.

The current study observed a negative impact on the QoL and loss of work productivity in patients, which was also observed in earlier Landmark surveys in the United States [13] and the UK [11, 12]. The Living with MPN patient survey, an extension of the MPN survey, substantiated the work productivity loss due to MPN, where 62.6% of the patients did not return to work [20], 30.2% of the patients had job termination, 24.8% went on medical disability leave, 21.8% had reduced work hours, 31.1% had an overall work impairment and 32.8% had activity impairment [21]. A similar trend for loss of work productivity was observed in 42% of patients, with them having to cut back approximately 12 h a week due to their condition. The impact of the disease is not restricted to patients, but also affected their employed caregiver and other family relations (11%) who had to reduce work hours and switch from full time to part time employment. Majority of the primary caregivers remained unemployed (89%). This contrasts with a similar study, which reported that 55.1% of patients required caregiver assistance, with many of the caregivers either choosing to reduce their work hours (26.3%) or having to quit their jobs (6.6%) [22].

Patients with MF across all risk categories and age groups in this study reported that emotional discomfort (92%) had an influence on daily living, while clinicians gave more significance to physical challenges. The “Back-to-life” initiative, a qualitative-quantitative survey, also confirmed the emotional struggles MF patients had and their need for greater disease understanding [23]. The difference in the opinions of patients and physicians indicated a gap of communication and a holistic approach towards a better management of the disease is the need of the hour. As per the physicians approximately 50% of the patients did not always agree with the primary treatment recommendation. Physicians considered an improvement in haemoglobin, along with reduction of spleen size as a successful therapy, whereas symptom improvement was the major success indicator for patients. Reduction of symptoms and a better QoL were considered the most important treatment goals by both physicians and patients in the MPN Landmark survey [12], whereas delaying of disease progression was the major treatment goal in the International MPN Landmark survey [13].

In the Indian context, doctors were cautious and avoided mentioning the disease’s progression as cancer, which hindered its management and affected patient expectations towards the condition. The MPN Landmark survey and survey of Taiwan also put forth the discordance in the physician and patient perceptions with regards to disease communication and treatment goals [12, 17]. According to the findings of the US MPN Landmark survey, patients were not aware of the symptoms they were experiencing, their doctor and patients did not share the same opinions about the goals of their treatment and patients were dissatisfied with their doctor’s overall management and communication [14].

The current study encompasses the symptomatology, treatment, QoL, work productivity measures and patient-physician communication alongside emphasising the gap between patient and physician perspectives, which is consistent with the results of the existing studies [14, 21]. Findings from our study suggest that managing disease burden in patients with MPNs is crucial to minimize disease impact on patient daily lives. A protracted delay between the development of symptoms and the beginning of treatment, as well as inadequate communication between patients and clinicians regarding symptoms and treatment management, are the most significant inadequacies observed in an Indian environment. Low satisfaction with treatment creates frustrations, nervousness and questions around their diagnosis and treatment choices.

It has been evinced that gaps exist in the physician and patients’ perceptions in terms of overall experience with MF, emotional disconnect, and consultation approach and frequency. Furthermore, patients considered symptom improvement as a success in the treatment of MF which was of lower priority for the physicians. The use of a standardized tool such as MPN-10 should be integrated in the routine clinical practice to evaluate the MF symptom burden. It is important to work towards understanding the variances in perceptions to provide the best treatment for MF. Keeping the patients informed of MF as a life-long and slow growing malignant condition can help patients have realistic expectations from disease outcome, therapy adherence, and need for regular investigations/ symptom tracking. Hence, there is a need for more holistic management of MF and a multidisciplinary approach to patient care and counselling.

The study also observed an average time lag of 9 months between the final diagnosis and the treatment initiation. It’s important to note that the time from diagnosis may not necessarily represent the actual disease latency, as there can be delays between disease onset and diagnosis. However, these findings indicate that patients diagnosed with MF may potentially benefit from early intervention, challenging the conventional ‘watch and wait’ approach often applied to newly diagnosed individuals [24].

## Limitations

The survey had a bias in patient selection, as physicians included only those patients with a specific level of education and/or financial resources that enabled them to comprehend and complete the survey. Moreover, the patients included in the survey might not be representative of the overall population of patients with MF, hence the result could not be generalized. In addition, majority of the physicians are from the private sector, with limited representation from government settings due to the inability of physicians to participate. The study does not delve into the in-depth analysis of epidemiological variation or past or present medical history, which significantly impacts the QoL parameters. Additionally, no independent panel validated the measurement instrument, thereby increasing the possibility of bias in outcome measurement. Patients’ self-reporting may have included some inconsistencies, which could be explained by ignorance and bias. Another limitation associated with the study is that no statistical tool was used to assure the survey’s reliability and confidence.

## Conclusion

In summary, the survey highlights the importance of understanding the symptom burden and the factors affecting the quality of life of individuals with MPNs. Such interventions play a pivotal role in mitigating the impact of the disease on patients’ daily routines. Furthermore, our results underscore the necessity for enhanced communication between patients and physicians, the establishment of standardized symptom monitoring procedures, and a shared understanding of treatment objectives.

## Electronic supplementary material

Below is the link to the electronic supplementary material.

Supplementary Material 1

## Data Availability

No datasets were generated or analysed during the current study.
